# Linkages between Plant Community Composition and Soil Microbial Diversity in Masson Pine Forests

**DOI:** 10.3390/plants12091750

**Published:** 2023-04-24

**Authors:** Jing Guo, Boliang Wei, Jinliang Liu, David M. Eissenstat, Shuisheng Yu, Xiaofei Gong, Jianguo Wu, Xiaoyong He, Mingjian Yu

**Affiliations:** 1 College of Life Sciences, Zhejiang University, Hangzhou 310058, China; guoj772@nenu.edu.cn (J.G.); 11707093@zju.edu.cn (B.W.); 2 College of Life and Environmental Science, Wenzhou University, Wenzhou 325035, China; jinliang.liu@foxmail.com; 3 Department of Ecosystem Science and Management, The Pennsylvania State University, University Park, State College, PA 16802, USA; dme9@psu.edu; 4 Ecological Forestry Development Center of Suichang County, Lishui 323300, China; schhc@sina.com (S.Y.); zjscgxf@sina.com (X.G.); 5 School of Life Sciences and School of Sustainability, Arizona State University, Tempe, AZ 85287, USA; jingle.wu@asu.edu; 6 Lishui Forestry Technology Promotion Station, Lishui 323000, China

**Keywords:** plant–microbe interactions, tree dominance, soil properties, microbial community, functional prediction

## Abstract

Plant species identity influences soil microbial communities directly by host specificity and root exudates, and indirectly by changing soil properties. As a native pioneer species common in early successional communities, Masson pine (*Pinus massoniana*) forests are widely distributed in subtropical China, and play a key role in improving ecosystem productivity. However, how pine forest composition, especially the dominance of plant functional groups, affects soil microbial diversity remains unclear. Here, we investigated linkages among woody plant composition, soil physicochemical properties, and microbial diversity in forests along a dominance gradient of Masson pine. Soil bacterial and fungal communities were mainly explained by woody plant community composition rather than by woody species alpha diversity, with the dominance of tree (without including shrub) species and ectomycorrhizal woody plant species accounting for more of the variation among microbial communities than pine dominance alone. Structural equation modeling revealed that bacterial diversity was associated with woody plant compositional variation via altered soil physicochemical properties, whereas fungal diversity was directly driven by woody plant composition. Bacterial functional groups involved in carbohydrate and amino acid metabolism were negatively correlated with the availability of soil nitrogen and phosphorus, whereas saprotrophic and pathogenic fungal groups showed negative correlations with the dominance of tree species. These findings indicate strong linkages between woody plant composition than soil microbial diversity; meanwhile, the high proportion of unexplained variability indicates great necessity of further definitive demonstration for better understanding of forest–microbe interactions and associated ecosystem processes.

## 1. Introduction

Soil bacteria and fungi live with plants to form complex biotic interactions, which play critical roles in nutrient cycle and carbon sequestration [1,2]. Soil microbial communities usually change a lot during forest secondary succession, while the drivers of variations on plant–microbe interaction are inconsistent among different studies [3,4]. Masson pine (*Pinus massoniana* Lamb.) forest is a common pioneer community following anthropogenic disturbance, and accounts for approximately 10% of forested area in China [5]. Masson pine is a favored species for soil and water conservation, carbon sequestration, and wood production [6,7]. Although significant differences in the composition and diversity of soil microbial communities between pine forests and other forest types have been widely reported [8,9], it is unclear how shifts in plant composition, such as relative dominance of plant functional groups, influence soil microbial diversity and function, especially in Masson pine forests.

Microbial communities in forest soils are simultaneously affected by many biotic and abiotic factors. Biotic factors such as tree species identity can regulate spatial variations in soil microbial communities at the regional scale via host specificity of symbiotic fungi, pathogenic fungi, and nitrogen-fixing bacteria [2,10,11]. Plant diversity is predicted to promote the diversification of soil microbes via diversified nutrient pools and plant hosts for microorganisms, as observed in temperate grasslands [12,13,14]. With regards to soil microbes in forests, evidence supporting a positive association between woody plant diversity and microbial diversity is mixed [15,16]. Alternatively, soil microorganisms may depend more on the abundance of key plant species than on plant diversity per se because of the host specificity of some microorganism groups [2,17,18]. Overall, effects of woody plants on microbial communities are often context dependent. Whether plant community composition has stronger effects than plant species alpha diversity on soil microbial communities needs further study.

Abiotic factors, such as soil pH and nutrient levels, have notable influence on microbial diversity [9,19,20]. Soil bacterial diversity may increase under conditions of neutral pH [19] and high nutrient availability [21]; these alterations in soil chemistry can be partly driven by variation in plant composition [22]. For example, plant functional traits tend to affect nutrient and organic carbon inputs into the soil by plant litter and root exudates, and lead to variations in soil acidity [11,23]. Comparatively, large groups of fungi (symbiotic and pathogenic fungi) are directly associated with specific host plants [2,24]. Exploring the direct and indirect effects of woody plants on soil microbial diversity is necessary to better understand plant–microbe interactions.

Plant and soil properties also affect microbial community composition and potential functions that are closely related to forest ecosystem processes [16,25,26]. For example, pine forest communities can easily recruit oligotrophic microorganisms because of the slow decomposition and limited nutrient release of dominant *Pinus* leaf litter [23,27,28]. Key functional groups involved in mycorrhizal symbiosis and amino-acid metabolism can be strongly affected by shifts in soil nutrient status and forest type [29,30]. Moreover, it has been demonstrated that there is strong competition between ectomycorrhizal (ECM) and saprotrophic fungi for soil resources [31]. In the canopy of forests in east Asia, the dominant trees are usually ECM species [32]. However, how microbial keystone taxa and functional groups are linked with plant community composition in Masson pine forests, a wide-spread community type in east Asia, is not known.

In this study, we examined soil bacteria and fungi along a gradient of pine dominance in Masson pine forests in eastern China. We obtained plot-scale information on geographic locations, plant community composition and diversity, soil physicochemical properties, and microbial diversity. Plant composition in our research represented not only visualized axes of principal coordinate analyses (PCoA) but also the relative dominance of specific functional group, such as tree (without including shrub) species and evergreen plant species. We addressed the question of how plant community influences the diversity and potential function of soil bacteria and fungi. We made the following predictions. (1) Variation in soil microbial composition and diversity would be more explained by plant community composition than by plant species diversity, because of the distinct litter chemistry, and litter and soil microbial structure among tree species [11,33]. (2) Bacterial diversity would be driven by plant composition via altered soil properties, whereas fungal diversity would be directly driven by plant composition due to the strong host specificity of mycorrhizal fungi [10,34]. (3) Saprotrophic and pathogenic fungal groups would be negatively correlated with the dominance of ECM plant species because of niche segregation with ECM fungi [31], while bacterial functional group would be mainly correlated with soil physicochemical properties.

## 2. Results

### 2.1. Description of Plant Community, Soil Physicochemical Properties, Bacteria and Fungi

Among 44 pine forest plots, woody plant species richness ranged among the plots from 6.0 to 21.0 and Shannon diversity ranged from 1.46 to 2.61. Soil maximum water holding capacity ranged from 291 g kg^−1^ to 726 g kg^−1^, pH ranged from 4.1 to 5.1, and nitrogen content ranged from 0.12% to 0.37% (Table 1). Study plots at three locations had similar range in pine dominance (PIV), woody plant alpha diversity, and soil physicochemical properties, while plant beta diversity (woody plant community composition) among locations differed. Thus, after taking plot location as a random effect factor, the wide variation in woody plant composition among plots allowed for analysis of their potential effects on soil microbial communities at the local scale. Moreover, we found a negative relationship between pine dominance (PIV) and woody plant alpha diversity (Figure S1 and Table S1). Plant PCoA1 was negatively correlated with tree species dominance (TIV, sum of importance values of all tree species) (Figure S1 and Table S2). Plant PCoA2 was positively correlated with the TIV and evergreen species dominance (EIV, sum of importance values of all evergreen woody species) (Figure S1 and Table S3).

According to soil samples at the quadrat level, a total of 11,923,709 high-quality bacterial reads were clustered into 2589 amplicon sequence variants (ASVs). At the plot level, the most dominant phylum in the bacterial community was *Acidobacteriota* (40.2% on average), followed by *Proteobacteria* (30.0%), *Actinobacteriota* (12.4%), *Verrucomicrobiota* (5.6%), and an unclassified phylum (4.2%). PICRUSt2 predicted that the main metabolic genes of bacteria were global and overview maps, metabolism of carbohydrates, amino acid, energy, cofactors and vitamins, etc. (Table S4). In total, 15,278,066 high-quality fungal reads were clustered into 2151 fungal ASVs. Phylum-level ASV characterization indicated that communities were dominated by *Basidiomycota* (47.3%) and *Ascomycota* (45.1%), followed by an unclassified phylum (4.4%). According to functional annotation of fungal ASVs by FungalTraits, functional groups of ectomycorrhizal, plant endophyte, soil saprotroph, and plant pathogen were relatively more abundant than other groups (Table S5).

### 2.2. Main Drivers of Soil Microbial Composition and Diversity

**Figure 1:**
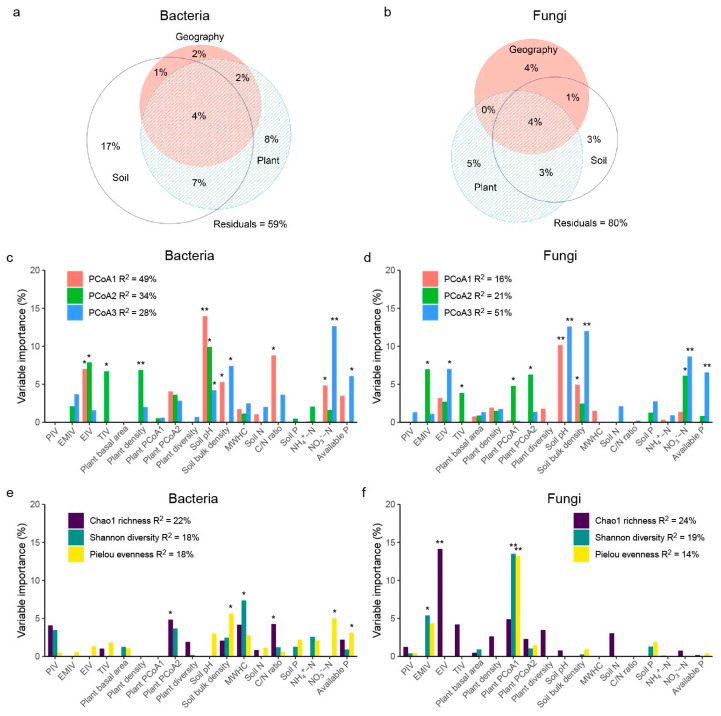
The fraction of the variation in the composition and alpha diversity of soil microbial communities explained by environmental predictors. (**a**,**b**) Variation partitioning analysis of geographic, plant, and soil environmental factors for the community variances of bacteria and fungi, with adjusted R^2^ displayed in the figure. Geographic factors contained longitude and latitude of each plot; plant factors contained PIV, EMIV, TIV, EIV, the basal area, density, PCoA1, PCoA2, and Shannon diversity of woody plant community; soil properties contained soil pH, soil bulk density, maximum water holding capacity, soil N, C/N ratio, soil P, NH_4_^+^ − N, NO_3_^−^ − N, and available P. (**c**–**f**) Random forest mean predictor importance of plant and soil environmental factors for the community variances of bacteria and fungi. PIV (importance values of Masson pine), Masson pine dominance; EMIV (sum of importance values of all ectomycorrhizal woody species), evergreen species dominance; EIV (sum of importance values of evergreen woody species), evergreen species dominance; TIV (sum of importance values of tree species), tree species dominance; MWHC, maximum water holding capacity. R^2^ values for bacterial community PCoA1, PCoA2, and PCoA3 axes are 23.05%, 15.09%, and 11.17%, respectively; R^2^ values for fungal community PCoA1, PCoA2, and PCoA3 axes are 17.64%, 7.78%, and 6.04%, respectively; R^2^ values for woody plant PCoA1 and PCoA2 axes are 31.84% and 16.74%, respectively. *, *p* < 0.05; **, *p* < 0.01.

In general, soil microbial communities were affected by both woody plant community composition and soil properties, but bacteria and fungi responded differently. Variation partitioning analysis showed that, overall, bacterial community composition was driven primarily by soil properties (29%), woody plant community composition (21%), and site location (9%), with 11% of the effect of soil factors shared with plant community factors (Figure 1a). Woody plant community traits explained 12% of overall fungal dissimilarities among plots, followed by soil properties (11%) and site location (9%), with 7% of the effect of plant community factors shared with the soil factors (Figure 1b).

We further analyzed the main explanatory variables of divergent microbial composition (beta diversity) and alpha diversity. Soil pH was the most important explanatory variable of bacterial composition dissimilarities, explaining 14%, 10%, and 4% of variance of PCoA1, PCoA2, and PCoA3, respectively. The dissimilarity of bacterial composition was also strongly explained by soil N and P availability, evergreen species dominance (EIV), and tree dominance without including shrub (TIV) (Figure 1c and Table S6). Soil maximum water holding capacity (MWHC) was the most important explanatory variable of bacterial alpha diversity, explaining 4%, 7%, and 3% of variance of Chao1 richness, Shannon diversity, and Pielou evenness, respectively (Figure 1e). For fungi, woody plant composition was the most important explanatory variable of fungal alpha diversity, with plant PCoA1 explaining 13% variation of fungal Shannon diversity and 13% variation of Pielou evenness, and EIV explaining 14% variation of fungal chao1 richness (Figure 1f). Dissimilarity of fungal composition was most strongly explained by soil pH and plant PCoA2 (Figure 1d). Comparatively, neither pine dominance nor plant alpha diversity showed strong links to variation in microbial community composition (Figure 1 and Table S6).

### 2.3. Relationship between Environmental Factors and Soil Microbial Diversity

**Figure 2:**
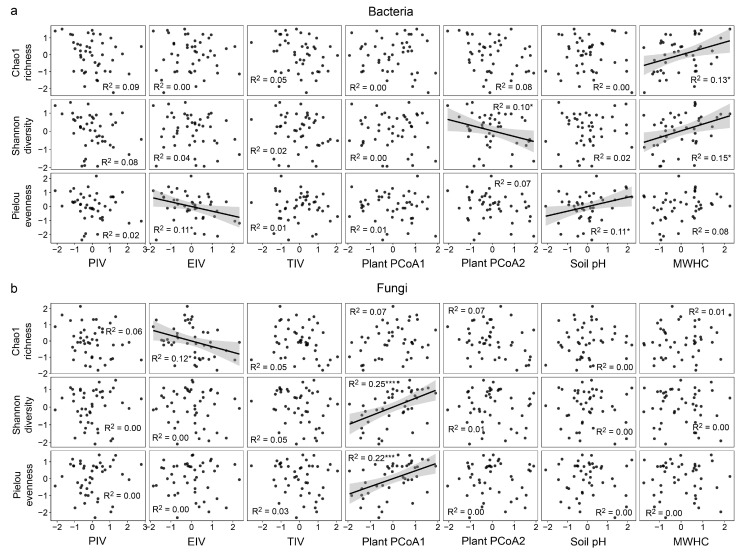
Pearson correlations between key environmental factors and soil bacterial (**a**) as well as fungal (**b**) alpha diversity. Values of all factors were shown after z-score transformation within each of the three counties. PIV, Masson pine dominance; EIV, evergreen species dominance; TIV, tree species dominance; MWHC, soil maximum water holding capacity. *, *p* < 0.05; ***, *p* < 0.001.

Soil bacterial and fungal alpha diversity were significantly associated with plant PCoA2 and PCoA1, respectively (Figure 2, Tables S7 and S8). Bacterial diversity was positively correlated with soil MWHC and pH (Figure 2a and Table S7). Fungal alpha diversity was negatively correlated with evergreen species dominance (EIV) but positively correlated with plant PCoA2 axis (Figure 2b). There were no significant correlations between fungal diversity and soil physicochemical properties (Figure 2b and Table S8). In addition, we found a negative relationship between plant PCoA2 and soil pH, and a negative relationship between pine dominance (PIV) and MWHC (Figure S1, Tables S9 and S10). There was no significant correlation between soil microbial diversity and PIV or between soil microbial diversity and overall tree dominance (TIV) (Figure S1, Tables S7 and S8).

**Figure 3:**
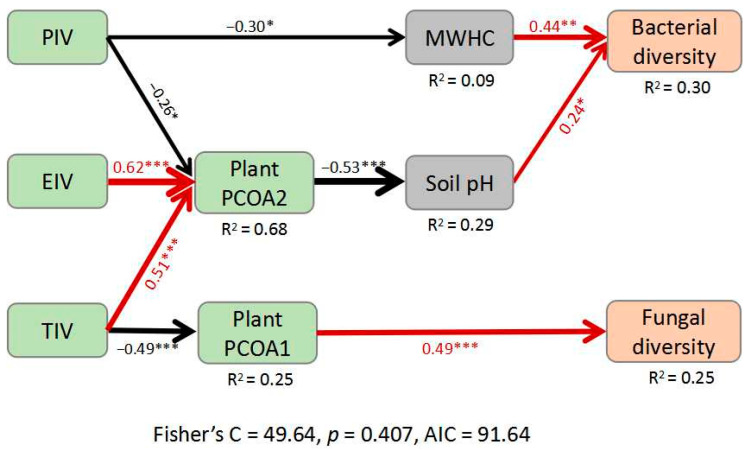
Structural equation model showing the pathways through which woody plant community traits influence the Shannon diversity of soil bacteria and fungi. Values associated with arrows mean standardized path coefficients. *, *p* < 0.05; **, *p* < 0.01; ***, *p* < 0.001. Red arrows indicate positive relationships, black arrows indicate negative relationships. Percentages associated with response variables represent the variance explained by the model. PIV, Masson pine dominance; EIV, evergreen species dominance; TIV, tree species dominance; MWHC, soil maximum water holding capacity.

We further used structural equation modeling (SEM) to link microbial Shannon diversity to plant composition and soil properties. SEM revealed that both bacterial and fungal diversity in the soil were driven by plant community composition, but with different mechanisms. Bacterial diversity was driven by plant composition via altered soil pH and MWHC, whereas fungal diversity was directly driven by woody plant composition (Figure 3).

### 2.4. Linkages of Microbial Composition and Functional Groups with Environmental Factors

**Figure 4:**
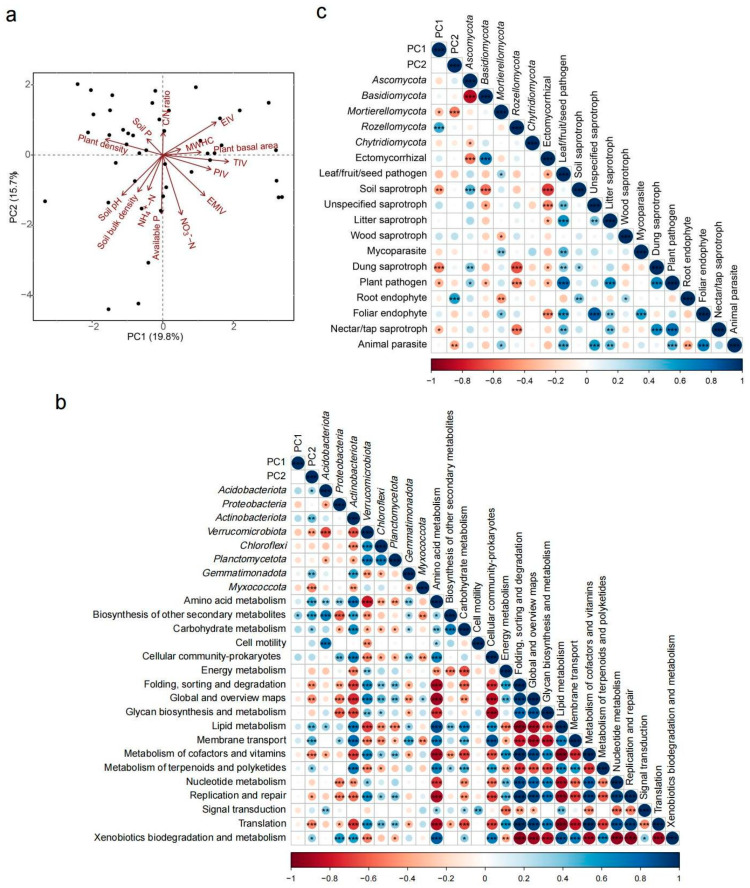
Relationship between environmental factors and taxonomic as well as functional groups of soil microorganisms. (**a**) Principal component analysis (PCA) for 14 environmental factors. PC1 axis was associated with environmental changes along tree dominance; PC2 axis was associated with soil resources. PIV, Masson pine dominance; EMIV, ECM woody species dominance; EIV, evergreen species dominance; TIV, tree species dominance; MWHC, soil maximum water holding capacity. (**b**) Pearson correlations between the relative abundance of phylum group and potential function genome of bacteria, and the first two environmental PCA axes. (**c**) Pearson correlations between the relative abundance of phylum and potential functional groups of fungi, and the first two environmental PCA axes. *, *p* < 0.05; **, *p* < 0.01; ***, *p* < 0.001.

The first PC axis (PC1) explained 19.8% of the variance and was positively correlated with overall tree dominance (TIV), evergreen dominance (EIV), and pine dominance (PIV), and negatively correlated with total plant density. Because PC1 axis was associated with environmental changes associated with shifts in tree dominance, we refer to PC1 as the “trees” axis (Figure 4a). The second PC axis (PC2) explained 15.7% of the variance and was negatively correlated with soil pH, available nutrient, and MWHC. Because these variables indicate soil resources, we refer to PC2 as the “soil” axis (Figure 4a).

The relative abundance of major bacterial phyla and predicted functional groups were mainly associated with soil properties. The phyla *Acidobacteriota*, *Actinobacteriota*, and *Gemmatimonadota* were positively correlated with PC2 and negatively correlated with soil pH. The phyla *Verrucomicrobiota* and *Myxococcota* were negatively correlated with PC2 and positively correlated with soil pH (Figure 4b and Figure S2a). Functional groups of amino acid, carbohydrate, and lipid metabolism were positively correlated with PC2, while functional groups of genetic information processing (including replication and repair, translation, folding, sorting, and degradation) were positively correlated with PC2 (Figure 4b).

For fungi, the relative abundance of major fungal phyla and predicted functional groups were mainly associated with plant composition (PC1). Fungal functional groups (i.e., ectomycorrhizal, saprotroph, and pathogen) were significantly correlated with the phyla *Ascomycota* and *Basidiomycota*, which were driven by plant PCoA1 (Figure 4c and Figure S2b). Moreover, we found a positive relationship between PC1 and *Rozellomycota*, and a negative relationship between PC1 and functional groups of saprotrophs and pathogens (Figure 4c). Functional groups of both saprotrophs and pathogens were negatively associated with the ectomycorrhizal group (Figure 4c).

## 3. Discussion

### 3.1. Soil Microbial Communities Are Mainly Associated with Woody Plant Composition Rather Than Diversity

Accumulating evidence suggests that soil microbial community composition is structured by tree species composition [10,11]. Plant species diversity is also an important factor influencing soil properties and microorganisms both across global terrestrial ecosystems and at the regional or local scale [3,35,36]. However, few studies focus on the role of plant functional groups on soil microbial community variation in forests [18,37,38]. Our results showed that soil microbial communities were mainly explained by woody plant community composition rather than alpha diversity, although there was a significantly negative relationship between plant diversity and pine dominance (PIV; Figure 1). This might be caused by the strong effects of tree species identity on the composition of both plant and microbial communities [10,39]. According to previous studies, the deciduous habit and/or leaf shape in trees determines litter acidity and microbial structure [33,40]. Soil microbial community structure and litter decomposition rate are also affected by vegetation type [9,41]. Supporting these results, both bacterial and fungal communities in our study were driven by the dominance (= total importance value) of tree species and of evergreen species in a plot. Thus, the differentiation of soil microbial communities in Masson pine forests is believed to be more related to plant composition than plant diversity.

### 3.2. Linkages between Plant Community Composition and Soil Microbial Diversity

We found that soil bacterial and fungal diversity were driven by the composition of woody plant communities through different pathways. More specifically, bacterial diversity was associated with plant compositional variation via altered soil properties, whereas fungal diversity was directly driven by plant composition (Figure 3). Probably because of variation in nutrient inputs by litter and root exudates of different plants [11,23,40], soil physicochemical properties in this study were significantly associated with woody plant compositional variation (Figure S1, Tables S9 and S10). Higher soil pH tended to negatively affect the relative abundance of certain bacterial phyla (i.e., *Acidobacteriota* and *Actinobacteriota*) but positively affected bacterial evenness and diversity (Figures S1 and S2 and Table S7). Similar results were drawn by Fierer and Jackson (2006) [19] and could be explained by the reduction of acidophilic bacteria under higher pH conditions [42]. Bacterial diversity tended to be positively associated with soil physical traits such as water holding capacity (Figure 2a), and this finding is generally in agreement with another study in weeping cypress plantations after the formation of forest gap where bacterial alpha diversity was positively correlated with soil moisture [43].

Consistent with other forest types [11], woody plant composition, rather than soil physicochemical properties, was significantly correlated with fungal diversity as well as the relative abundance of dominant phyla in this Masson pine forest study (Figure 2b and Figure S2b). This result is not surprising, considering the strong host specificity of mycorrhizal fungi [10,34]. Furthermore, fungi are more likely to be influenced by litter origin and chemistry, as they can degrade complex carbon compounds [31,44]. In contrast, in some other pine forest studies, soil fungal communities were more strongly influenced by soil physicochemical properties than by forest structural variables [16,27]. Further definitive demonstration is necessary to clarify the relationship between plant community composition and soil microbiomes behind these observations.

### 3.3. Linkages between Plant Community Composition and Microbial Functional Groups

A large group of fungi are obligate root symbionts, which strengthen fungal host specificity [2,10,34]. Although there are limitations to functional prediction of microbial communities, we use this approach to explore possible linkages between plant community composition and microbial functional groups. Based on the functional prediction of fungal guilds by FungalTraits, the relative abundance of ectomycorrhizal (ECM) fungi was positively correlated with the *Basidiomycota* phylum, and ECM fungi was also indirectly driven by woody plant composition (Figure 4 and Figure S2b). In addition, our results showed different environmental preferences between ectomycorrhizal and saprotrophic fungi. This finding was in agreement with previous reports [45,46], and it supports a competing interaction phenomenon known as the ‘Gadgil effect’, which affects the roles of these two fungal guilds in the breakdown and recycling of soil organic matter [31,47,48]. Probably due to the greater resistance against pathogens provided by Hartig net and fungal mantle surrounding fine roots of ECM trees [49], pathogenic fungi in this study were negatively correlated with both ectomycorrhizal fungi and tree dominance (Figure 4). Therefore, in Masson pine forests where the canopy is mainly dominated by ectomycorrhizal trees, the niche segregation of saprotrophic, pathogenic, and ectomycorrhizal fungi can be reasonably predicted by variation in tree species dominance.

PICRUSt2 was used to predict bacterial gene abundance within metabolic pathways. Results of Pearson correlation on bacterial functional group with environmental variables indicated the potential association between bacterial functional groups and soil physicochemical properties. Like previous research [50], lower soil nutrient availability and pH in this study strengthened the pathways of amino acid, carbohydrate, and lipid metabolism (Figure 4). In infertile soil, we conclude that bacterial metabolic function was mainly associated with nutrient metabolism, especially carbohydrate metabolism, for resource acquisition [51]. Moreover, bacteria can secrete a variety of enzymes for strong degradation of complex and diverse resources by changing the potential “amino acid metabolism” function [51]. We also found that bacterial groups involved in genetic information processing showed positive relationships with soil nutrient availability and pH (Figure 4), and the overall functional prediction suggested that in common with bacterial structure, bacterial functions tend to be driven by soil physicochemical properties rather than by plant composition.

## 4. Material and Methods

### 4.1. Study Area

**Figure 5:**
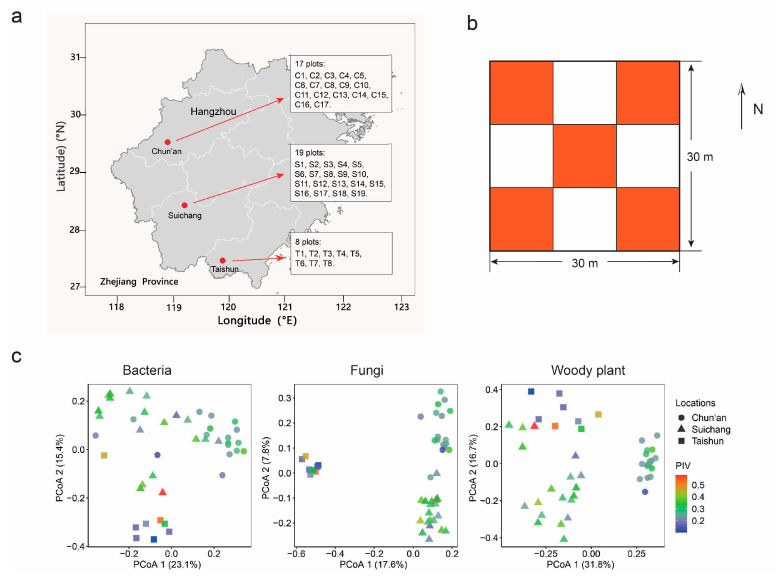
The location, sampling design, and ordination of the bacteria, fungi, and woody plant by principal coordinate analysis (PCoA) at plot level. (**a**) The locations of the 44 study plots in Zhejiang province, China. (**b**) Five, 10 m × 10 m quadrats (red color) were investigated at each plot. (**c**) Both soil microbiomes and woody plants are separable by three locations. PCoA was based on the Bray–Curtis distance matrices. Samples are shaped according to site locations and colored according to the importance value of Masson pine (PIV).

Natural Masson pine forests are widely distributed in southern China, covering 5.5 million ha, comprising about 4.5% of the national total area of natural arbor forest [52]. The study was conducted in Zhejiang province, southeastern China, characterized by a monsoon climate, with local mean annual temperatures of 16.2–17.2 °C, and mean annual precipitations of 1515–2047 mm (China Meteorological Data Service Centre, http://data.cma.cn/ (accessed on 10 June 2022)). The forest sites were located from 27°32′58″ to 29°33′51″N latitude, 118°41′22″ to 119°46′55″E longitude (Figure 5a and Table S11). Basic location, elevation, plant community traits, and soil properties of three locations (Chun’an, Suichang, and Taishun County) are described in Table 1.

### 4.2. Plant Community Investigation

Forty-four Masson pine forest plots (30 m × 30 m each) were established in 2020 and 2021. All plots were located at least 100 m away from the edge of non-forest land. The study sites had been previously subjected to similar management practices. These forests developed from secondary succession of cleared evergreen broad-leaved forests following logging in the 1950s. Now, Masson pine is a common species in these plots. At each plot, a GPS global positioning system was used to measure the longitude and latitude information. A total of five 10 m × 10 m quadrats (one at the center and four in each plot corner) were used for plant and soil investigation. After investigating at the quadrat level, the five replicates were averaged to obtain plot-level estimates of plant and soil microbial communities, and soil physicochemical properties, for each 30 m × 30 m plot (Figure 5b,c).

The number, diameter, and species for all free-standing woody plants with diameter at breast height (i.e., 1.3 m above the ground) ≥1 cm were measured to calculate plant density, basal area, and frequency information. The Shannon–Wiener index and species richness were calculated to estimate species alpha diversity [53,54]:

$$\mathrm{Shannon}–\mathrm{Wiener}\;\mathrm{Index}\;(H)\;H=-\sum_{i=1}^{s}\;P_{i}\;\ln\;P_{i}$$

where *s* is the total species number of the community, and *P_i_* is the relative importance value of species *i*.

Importance value (IV) was calculated to estimate the relative dominance property of each species [55]:

$$\mathrm{IV}=\frac{\;\mathrm{relative}\;\mathrm{density}\;+\;\mathrm{relative}\;\mathrm{basal}\;\mathrm{area}\;+\;\mathrm{relative}\;\mathrm{frequency}}{3}.$$

### 4.3. Dominance of Plant Functional Groups

The mycorrhizal type of each woody plant species was assigned based on the morphological criteria according to published information [56,57]. When mycorrhizal type was not available at the plant species level, these plants were categorized according to the mycorrhizal type of other species within the same genus [58]. In total, we had 20 ectomycorrhizal woody plant species and 124 woody plant species of other mycorrhizal types (including arbuscular mycorrhizal, ericoid mycorrhizal, and non-mycorrhizal type) from 44 plots (Table S12), with 20.3% of species mycorrhizal type being categorized based on genus level. The dominance of ECM plant species (EMIV) was calculated by summing the importance values for all ECM species in a quadrat. Additionally, we quantified leaf habit (evergreen or deciduous) and growth form (tree or shrub) to explore the compositional divergence among plots. The relative dominance of evergreen species (EIV) was calculated by summing the importance values for all evergreen species (including trees and shrubs) in a quadrat. The relative dominance of tree species (TIV) was calculated by summing the importance values for all tree species (without including shrub species) in a quadrat. The sum of the importance values of all woody plant species (including trees and shrubs) in a quadrat was 100%.

### 4.4. Soil Sampling and Physicochemical Analyses

Soil samples were collected in May 2020 in Chun’an and Suichang County, and June 2021 in Taishun County. One 100-cm^3^ core was randomly collected at the center of each quadrat for the measurement of soil bulk density and maximum water holding capacity (MWHC). Three 0–10 cm deep soil cores were randomly collected around the center of each quadrat after removing the litter layer, mixing and passing through a sieve (2-mm mesh size) to form one soil sample. A total of 220 soil samples (5 quadrat samples × 44 plots) were collected. Each sieved sample was separated into three subsamples. One part was stored at −80 °C for DNA extraction and sequencing. The second part was stored at 4 °C for measurement of extractable soil ammonium and nitrate nitrogen (N) within 72 h. The third part was air-dried for the measurement of other soil chemical properties. All laboratory analyses were completed within two months of field sampling.

A total of nine soil physicochemical variables were measured in this study. Soil bulk density (g cm^−3^) and maximum water holding capacity (MWHC, g kg^−1^) were measured using the cutting ring method (LY/T 1215–1999) [59]. Soil pH was measured using a pH meter (SevenEasy S20K, Mettler Toledo, Greifensee, Switzerland), with water and soil in a 2.5:1 ratio by mass. Soil carbon (Soil C, mg g^−1^) and N concentrations (Soil N, mg g^−1^) were determined with an elemental analyzer (Vario MACRO Cube, Elementar, Langenselbold, Germany) after grinding and passing through a sieve (0.15-mm mesh size). Soil ammonium N (NH_4_^+^−N, mg kg^−1^) and nitrate N (NO_3_^−^−N, mg kg^−1^) were quantified using continuous flow analyzer (San++, Skalar, Breda, Holland) after KCl (2 mol/L) extraction. Soil phosphorus (Soil P, mg g^−1^) was digested with sulfuric acid-perchloric acid after grinding and passing through a sieve (0.15-mm mesh size). Soil available P (mg kg^−1^) was extracted with ammonium fluoride and hydrochloric acid [60]. Soil P and available P were both quantified by inductively coupled plasma optical emission spectrometry (Optima 8300, PerkinElmer, Waltham, MA, USA).

### 4.5. DNA Extraction and Sequencing

Soil DNA was extracted from samples using the TGuide S96 Magnetic Soil /Stool DNA Kit (Tiangen Biotech (Beijing) Co., Ltd., Beijing, China), following the manufacturer’s protocol. The DNA concentration of the samples was measured using the Qubit dsDNA HS Assay Kit and Qubit 4.0 Fluorometer (Invitrogen, Thermo Fisher Scientific, Hillsboro, ON, USA). For bacteria, the V3-V4 region of 16S rRNA gene was targeted with primer pair 338F (5′-ACTCCTACGGGAGGCAGCA-3′) and 806R (5′-GGACTACHVGGGTWTCTAAT-3′) [61,62], with the expected amplicon size of 450bp. For fungi, the internal transcribed spacer 1 (ITS1) region of rRNA gene was amplified using primers ITS1F (5′-CTTGGTCATTTAGAGGAAGTAA-3′) and ITS2R (5′-GCTGCGTTCTTCATCGATGC-3′) [62,63], with the expected amplicon size of 400 bp. PCR amplification was performed for both the 16S rRNA gene and the ITS1 region, with reactions containing 5–50 ng DNA template, 0.3 μL (10 μM) of each primer, 5 μL KOD FX Neo Buffer, 2 μL (2 mM each) dNTP, 0.2 μL KOD FX Neo, and up to 10 μL ddH2O. For the V3-V4 region of 16S rRNA gene, thermal cycling conditions were as follows: an initial denaturation at 95 °C for 5 min, followed by 25 cycles of denaturation at 95 °C for 30 s, annealing at 50 °C for 30 s, and extension at 72 °C for 40 s, and a final step at 72 °C for 7 min. Thermal cycling conditions of ITS1 gene were as follows: an initial denaturation at 95 °C for 5 min, followed by 25 cycles of denaturation at 95 °C for 1 min, annealing at 50 °C for 30 s, and extension at 72 °C for 1 min, and a final step at 72 °C for 7 min. All PCR amplicons were purified with Agencourt AMPure XP Beads (Beckman Coulter, Indianapolis, IN, USA) and quantified using the Qubit dsDNA HS Assay Kit and Qubit 4.0 Fluorometer (Invitrogen, Thermo Fisher Scientific, Hillsboro, ON, USA). High-throughput sequencing of rRNA genes of bacteria and fungi were analyzed using the Illumina NovaSeq 6000 PE250 platform (Illumina, Santiago, CA, USA) at Biomarker Technologies Corporation, Beijing, China.

### 4.6. Bioinformatics

Raw data were primarily filtered by Trimmomatic v0.33 [64]. Identification and removal of primer sequences was processed by Cutadapt v1.9.1 [65]. The remaining high-quality sequences were processed using QIIME 2 (v. 2020.6) [66], in which the reads were processed and applied to DADA2 pipeline for the assignment of amplicon sequence variants (ASVs) [67]. The sequences were classified to ASVs by naive Bayesian classifier-based method, with 0.005% conservative threshold for ASV filtration [68]. Species annotation was processed with classify-sklearn in QIIME2 software. To determine the taxonomic classification of each ASV of bacteria and fungi, we searched, respectively, the databases of SILVA 138.1 [69] and UNITE 8.0 [70]. ASVs that were not classified into bacteria or fungi were removed. Before downstream analysis, bacterial samples were rarefied to 15,182 sequences per sample, with one sample below this threshold that was removed from analysis. Fungal samples were rarefied to 41,023 sequences per sample, with two samples below this threshold that were removed from analysis. Both community composition and diversity analyses were carried out on ASV tables. Ecological functions of the soil bacteria and fungi were assigned, respectively, by PICRUSt2 v2.3.0 [71] and FungalTraits v0.0.3 [72].

### 4.7. Statistical Analyses

All statistical analyses and visualizations were performed in the R software environment (v4.2.1, R Development Core Team). Alpha diversity indices (Chao1 richness, Shannon diversity, and Pielou evenness) of bacteria and fungi were calculated from each of the 220 (44 plots × 5 quadrats) samples. All information from five replicate quadrats were averaged to obtain 30 m × 30 m plot-level estimates of plant communities, soil microbial communities, and soil physicochemical properties. Principal coordinate analysis (PCoA) based on the Bray–Curtis distance matrices was conducted using the vegan package v2.6-2 [73] to visualize the variation in plant, bacterial, and fungal community structure. During the following statistical analyses, z-score transformation was used to standardize the environmental data, with an overall mean of 0 and standard deviation of 1. For variation partitioning analysis and principal component analysis which require low collinearity on environmental data, variance inflation factor (VIF) was analyzed to remove collinear variables within a multiple regression until all remaining VIFs were below 4 [74,75]. Because there were significant differences in plant community and soil properties among three locations (Figure 5c and Table 1), random effects from plot location were taken into account on partial Mantel test and linear mixed effect model. In the following analyses (i.e., random forest model, Pearson correlation, structural equation model, and principal component analysis), all environmental data and microbial diversity data within each location were z-score transformed separately, and all microbial compositional data on phylum taxa and functional groups within each location were centered log-ratio transformed in order to remove location effects, and meanwhile to meet the assumption of normality and homogeneity of variance for the following analysis of Pearson correlation and structural equation model.

To test the first prediction, we used the partial Mantel test to reveal the effects of woody plant composition and diversity, and soil properties on microbial community composition, while controlling for the effect of plot geography. We used variation partitioning analysis and Monte Carlo permutation tests (999 permutations) to determine the relative importance and significance of geography (i.e., longitude and latitude), plant community (i.e., PIV, EMIV, TIV, EIV, plant basal area, plant density, plant PCoA1, plant PCoA2, and Shannon diversity), and soil properties (i.e., soil pH, bulk density, MWHC, soil N, C/N ratio, soil P, NH_4_^+^ − N, NO_3_^−^ − N, and available P) on the variation in microbial community composition. Partial Mantel tests and variation partitioning analysis were conducted using the vegan package v2.6-2 [56]. Percentage increases in the mean squared error (MSE) of variables in a random forest model were used to estimate the importance of plant composition and soil physicochemical factors to microbial composition and diversity [76]. Random forest model was performed using the randomForest package v4.6-14 and rfPermute package v2.5.

To test the second prediction, Pearson correlation was performed using the Hmisc package v4.6-0 and psych package v2.1.9 to check the relationship between environmental factors and soil microbial diversity. A linear mixed effect model was performed using the lmerTest package v.3.1-3.to conduct the analyses of variances for soil microbial diversity (Shannon diversity), with plant community and soil property as fixed factors, and plot location as a random factor. The influence of plot location on the linkage between key environmental factors (such as plant composition and soil acidity) and soil microbial diversity was ruled out during the selection of optimum linear mixed effect model. Structural equation modeling was also performed using the piecewiseSEM package v4.0.5 to estimate the synthetic effect pathways of plant composition on microbial diversity.

To test the third prediction, the relative abundance of bacteria and fungi was assigned at the phylum and functional levels. A principal component analysis (PCA) was performed on 14 environmental variables to reduce the number of environmental variables, and the first 2 principal components (PC1 and PC2) explained 35.5% of the total variance (Table S13). The PC1 axis was associated with high dominance of trees without including shrubs and high dominance of ECM plant species. The PC2 axis was associated with low soil nutrient availability and lower pH value. We used Pearson correlation analysis to check the relationship between environmental variables and the relative abundance of phyla as well as functional groups of bacteria and fungi.

## 5. Conclusions

Plant–microbe interactions are complex and poorly understood at the community level. Our results suggest that soil bacteria and fungi in Masson pine forests are influenced mainly by woody plant species composition, rather than by woody plant species diversity. The relative dominance of tree species and of ECM woody plant species explained microbial community variation better than simply pine dominance. Bacterial diversity was indirectly affected by woody plant composition via altered soil pH, while fungal diversity was directly associated with plant composition. According to results derived using a functional prediction approach, the relative abundance of functional groups of bacteria and fungi was mainly associated with soil available nutrients and plant community composition, respectively. Bacterial functional groups involved in carbohydrate and amino acid metabolism seem to be increased in soil environments with low available nutrients, while saprotrophic and pathogenic fungal groups were negatively driven by increases in tree dominance. Taken together, our results indicate more important effects of the species identity and functional traits of woody plants than those of general plant species diversity on soil microbial diversity. Because of the high proportion of unexplained variability in this field study, further research should focus on more definitive demonstration of how plant community composition affects soil microbiomes, and the interaction between pine dominance and site-specific effects should be taken into consideration in plant–microbe interactions of pine forests.

## Figures and Tables

**Table 1:** Geographic location, altitude, woody plant community, and soil variables of each location in southeastern China. PIV (importance values of Masson pine), Masson pine dominance; EIV (sum of importance values of all evergreen woody species), evergreen species dominance; TIV (sum of importance values of tree species without including shrubs), tree species dominance. MWHC, maximum water holding capacity.

Parameters	Chun’an	Suichang	Taishun
Latitude (N)	29°29′26″–29°33′51″	28°35′34″–28°37′11″	27°33′48″–27°40′36″
Longitude (E)	118°41′22″–118°59′09″	118°56′08″–119°28′02″	119°41′56″–119°46′55″
Altitude (m a.s.l.)	112–257	228–641	221–602
PIV	0.19–0.37	0.19–0.43	0.10–0.55
EIV	0.63–0.94	0.47–0.95	0.83–0.95
TIV	0.26–0.47	0.53–0.85	0.39–0.71
Plant species richness	7.4–17.0	6.0–21.0	7.2–18.6
Plant Shannon diversity	1.5–2.2	1.2–2.6	1.4–2.5
Soil MWHC (g kg^−1^)	291–672	326–525	347–726
Soil pH	4.1–5.1	4.2–5.1	4.2–5.0
Soil C (mg g^−1^)	15.2–42.6	23.8–39.1	23.8–61.7
Soil N (mg g^−1^)	1.2–3.0	1.5–2.8	1.6–3.7
Soil P (mg g^−1^)	0.13–0.40	0.10–0.28	0.13–0.27
C/N ratio	11.9–16.5	12.8–17.3	14.4–18.1

## Data Availability

The raw sequences of bacteria and fungi were submitted to the NCBI-SRA and are available under the accession number PRJNA836363 and PRJNA836392, respectively.

## Supplementary Materials

The following supporting information can be downloaded at: https://www.mdpi.com/article/10.3390/plants12091750/s1. Figure S1: Pearson correlations between environmental variables and soil bacterial and fungal alpha diversity; Figure S2: Pearson correlations between environmental factors and the relative abundance of bacterial phylum taxa (a) and fungal phylum taxa (b); Table S1: Results of linear mixed-effects models for plant Shannon diversity using Masson pine dominance (PIV, importance value of Masson pine), ECM plant species dominance (EMIV, sum of importance values of ectomycorrhizal woody species), evergreen species dominance (EIV, sum of importance values of all evergreen woody species) and tree species dominance (TIV, sum of importance values of tree species) as fixed factors; Table S2: Results of linear mixed-effects models for plant PCoA1 using Masson pine dominance (PIV), ECM plant species dominance (EMIV), evergreen species dominance (EIV) and tree species dominance (TIV) as fixed factors; Table S3: Results of linear mixed-effects models for plant PCoA2 using Masson pine dominance (PIV), ECM plant species dominance (EMIV), evergreen species dominance (EIV) and tree species dominance (TIV) as fixed factors; Table S4: Relative abundance of bacterial phylum and functional taxa in all samples; Table S5: Relative abundance of fungal phylum and functional taxa in all samples; Table S6: Results of the partial Mantel test showing differences in Bray-Curtis distances of the soil microbial communities due to woody plant community and soil properties; Table S7: Results of linear mixed-effects models for soil bacterial diversity (Shannon diversity) using plant community traits and soil properties as fixed factors separately; Table S8: Results of linear mixed-effects models for soil fungal diversity (Shannon diversity) using plant community traits and soil properties as fixed factors separately; Table S9: Results of linear mixed-effects models for soil pH using Masson pine dominance (PIV), ECM woody species dominance (EMIV), plant PCoA1, plant PCoA2 and plant Shannon diversity as fixed factors; Table S10: Results of linear mixed-effects models for soil maximum water holding capacity (MWHC) using Masson pine dominance (PIV), ECM plant species dominance (EMIV), plant PCoA1, plant PCoA2 and plant Shannon diversity as fixed factors; Table S11: Characteristics of the geographic location and woody plant community of the 44 Masson pine forest plots in southeastern China; Table S12: Mycorrhizal type, leaf habit and growth form of woody plants appeared in 44 plots; Table S13: The first two axes values of Principal component analysis (PC1 and PC2) for 14 environmental factors.
