# Supplementary material for: Linkages between Plant Community Composition and Soil Microbial Diversity in Masson Pine Forests
Source: Plants (Basel). 2023 Apr 24;12(9):1750. doi: 10.3390/plants12091750 (PMC10181205; doi:10.3390/plants12091750)
Supplement: Supplementary file 1 [file plants-12-01750-s001.zip › plants-2325823-supplementary.pdf]

Supplemental materials

Linkages between Plant Community Composition and Soil Microbial Diversity in Masson Pine Forests

Jing Guo [1], Boliang Wei [1], Jinliang Liu [2], David M. Eissenstat [3], Shuisheng Yu [4], Xiaofei Gong [4], Jianguo Wu [5], Xiaoyong He [6,*] and Mingjian Yu [1,*]

* Correspondence: lshexy@hotmail.com (X.H.); fishmj@zju.edu.cn (M.Y.)

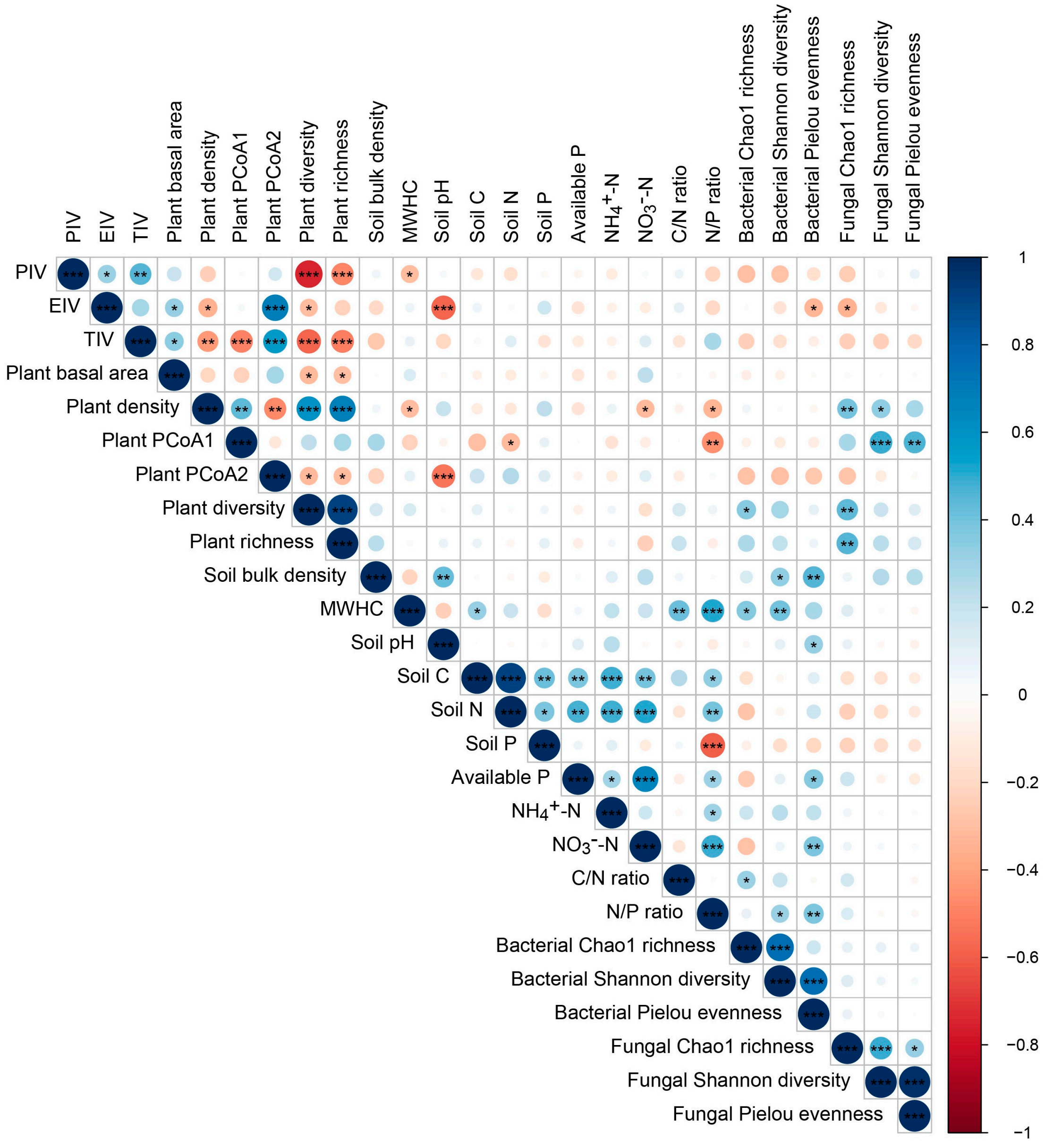

Figure S1. Pearson correlations between environmental variables and soil bacterial and fungal alpha diversity. PIV (importance values of Masson pine), Masson pine dominance; EIV (sum of importance values of all evergreen woody species), evergreen species dominance; TIV (sum of importance values of all tree species), tree species dominance; MWHC, soil maximum water holding capacity. *, $P < 0.05$; **, $P < 0.01$; ***, $P < 0.001$.

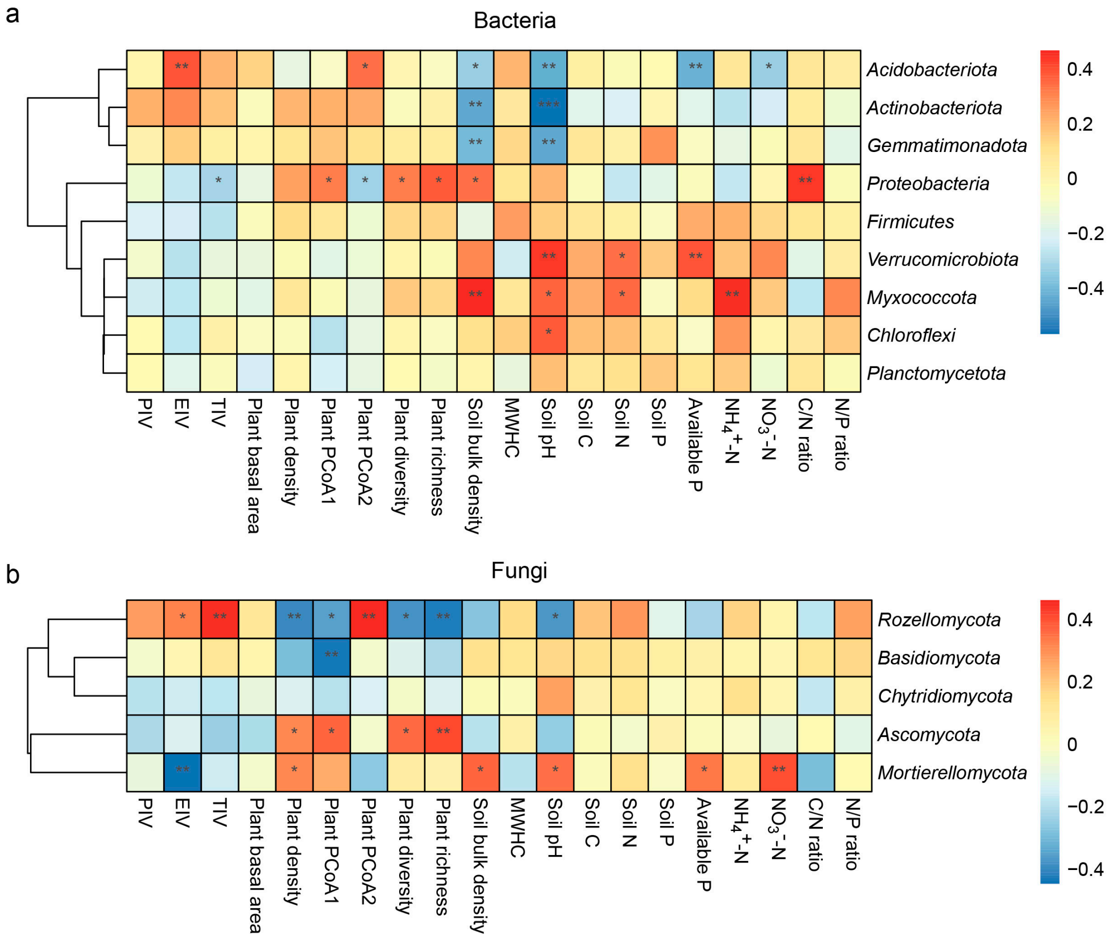

Figure S2. Pearson correlations between environmental factors and the relative abundance of bacterial phylum taxa (a) and fungal phylum taxa (b). PIV, Masson pine dominance; EIV, evergreen species dominance; TIV, tree species dominance; MWHC, soil maximum water holding capacity. *, $P < 0.05$; **, $P < 0.01$; ***, $P < 0.000$.

Table S1. Results of linear mixed-effects models for plant Shannon diversity using Masson pine dominance (PIV, importance value of Masson pine), ECM plant species dominance (EMIV, sum of importance values of ectomycorrhizal woody species), evergreen species dominance (EIV, sum of importance values of all evergreen woody species) and tree species dominance (TIV, sum of importance values of tree species) as fixed factors. Impact factors in bold type were significant at $P < 0.05$.

Impact factors	Estimate	SE	T	*P*
**PIV**	**-0.570**	**0.112**	**-5.057**	**< 0.001**
EMIV	-0.154	0.117	-1.316	0.195
EIV	-0.064	0.112	-0.573	0.569
**TIV**	**-0.559**	**0.151**	**-3.704**	**< 0.001**

Table S2. Results of linear mixed-effects models for plant PCoA1 using Masson pine dominance (PIV), ECM plant species dominance (EMIV), evergreen species dominance (EIV) and tree species dominance (TIV) as fixed factors. Impact factor in bold type was significant at $P < 0.05$.

**Impact factors**	**Estimate**	**SE**	**T**	***P***
PIV	0.052	0.050	1.039	0.305
**EMIV**	**-0.216**	**0.053**	**-4.054**	**< 0.001**
EIV	-0.071	0.051	-1.391	0.172
**TIV**	**0.489**	**0.069**	**7.008**	**< 0.001**

Table S3. Results of linear mixed-effects models for plant PCoA2 using Masson pine dominance (PIV), ECM plant species dominance (EMIV), evergreen species dominance (EIV) and tree species dominance (TIV) as fixed factors. Impact factor in bold type was significant at $P < 0.05$.

**Impact factors**	**Estimate**	**SE**	**T**	***P***
PIV	-0.133	0.081	-1.641	0.109
**EMIV**	**0.222**	**0.085**	**2.611**	**0.012**
**EIV**	**0.609**	**0.081**	**7.487**	**< 0.001**
**TIV**	**0.428**	**0.110**	**3.893**	**< 0.001**

Table S4. Relative abundance of bacterial phylum and functional taxa in all samples.

Phylum taxa	Relative abundance (%)	Functional taxa	Relative abundance (%)
Actinobacteriota	40.2	Global and overview maps	42.1
Proteobacteria	30.0	Carbohydrate metabolism	8.9
Acidobacteriota	12.4	Amino acid metabolism	7.5
Verrucomicrobiota	5.6	Energy metabolism	4.3
Unclassified	4.2	Metabolism of cofactors and vitamins	4.0
Chloroflexi	2.3	Membrane transport	3.4
Planctomycetota	1.2	Nucleotide metabolism	2.9
Gemmatimonadotaes	1.1	Translation	2.8
Myxococcota	1.1	Signal transduction	2.6
Firmicutes	0.2	Lipid metabolism	2.5
Others	1.7	Replication and repair	2.3
		Xenobiotics biodegradation and metabolism	1.9
		Cellular community - prokaryotes	1.7
		Metabolism of other amino acids	1.6
		Folding, sorting and degradation	1.4
		Metabolism of terpenoids and polyketides	1.2
		Cell motility	1.2
		Glycan biosynthesis and polyketides metabolism	1.1
		Biosynthesis of other secondary metabolites	1.0
		Others	5.6

Table S5. Relative abundance of fungal phylum and functional taxa in all samples.

Phylum taxa	Relative abundance (%)	Functional taxa	Relative abundance (%)
Basidiomycota	47.3	Ectomycorrhizal	29.4
Ascomycota	45.1	Foliar endophyte	15.8
Unclassified	4.4	Soil saprotroph	13.1
Mortierellomycota	2.0	Root endophyte	12.1
Rozellomycota	0.7	Unspecified saprotroph	6.0
Chytridiomycota	0.3	Litter saprotroph	5.2
Others	0.2	Leaf/fruit/seed pathogen	4.3
		Plant pathogen	3.1
		Animal parasite	2.9
		Wood saprotroph	2.3
		Animal parasite	1.9
		Mycoparasite	1.5
		Dung saprotroph	1.0
		Others	1.4

Table S6. Results of the partial Mantel test showing differences in Bray-Curtis distances of the soil microbial communities due to woody plant community and soil properties. The partial Mantel test controlled the effect of the location matrix of plots, including longitude and latitude. Impact factors in bold type were significant at $P < 0.05$. PIV, Masson pine dominance; EMIV, ECM woody species dominance; EIV, evergreen species dominance; TIV, tree species dominance; MWHC, soil maximum water holding capacity.

**Impact factors**	**Bacteria**		**Fungi**	
	**R**	***P***	**R**	***P***
Woody plant community				
**Plant PCoA1**	**0.136**	**0.009**	-0.047	0.980
**Plant PCoA2**	**0.274**	**0.009**	**0.406**	**0.004**
Plant species richness	-0.043	0.889	0.045	0.271
Plant shannon diversity	-0.035	0.889	0.000	0.683
Total basal area	0.088	0.091	-0.034	0.907
**Total density**	0.019	0.458	0.074	0.119
EMIV	0.061	0.219	0.061	0.183
PIV	0.037	0.312	0.031	0.382
**EIV**	**0.290**	**0.001**	**0.273**	**0.004**
TIV	0.127	0.064	-0.126	0.993
Soil properties				
**Soil bulk density**	**0.339**	**0.001**	-0.033	0.907
**MWHC**	**0.196**	**0.003**	**0.169**	**0.004**
**pH**	**0.377**	**0.003**	**0.182**	**0.004**
**Soil C**	**0.189**	**0.019**	**0.189**	**0.004**
**Soil N**	**0.222**	**0.003**	**0.161**	**0.010**
Soil P	0.070	0.176	-0.009	0.712
**C/N ratio**	0.082	0.168	0.080	0.119
**N/P ratio**	**0.159**	**0.014**	0.085	0.119
**Available P**	**0.228**	**0.007**	0.001	0.683
NH_4^+–N	-0.053	0.889	-0.098	0.988
**NO_3^-–N**	**0.270**	**0.003**	**0.117**	**0.048**

Table S7. Results of linear mixed-effects models for soil bacterial diversity (Shannon diversity) using plant community traits and soil properties as fixed factors separately. Plant community traits contained Masson pine dominance (PIV), ECM woody species dominance (EMIV), evergreen species dominance (EIV), all tree species dominance (TIV), plant PCoA1, plant PCoA2 and plant Shannon diversity. Soil properties included soil pH, maximum water holding capacity (MWHC), soil C/N ratio, soil N, soil P and available P. Impact factors in bold type were significant at $P < 0.05$.

**Impact factors**	**Estimate**	**SE**	**T**	***P***
Plant factors				
PIV	-0.116	0.261	-0.445	0.695
EMIV	0.174	0.278	0.626	0.535
EIV	0.098	0.319	0.309	0.759
TIV	-0.120	0.417	-0.289	0.774
Plant PCoA1	0.587	0.571	1.029	0.324
Plant PCoA2	-0.309	0.381	-0.811	0.424
Plant diversity	0.162	0.282	0.575	0.570
Soil factors				
**MWNC**	**0.592**	**0.125**	**4.728**	**< 0.001**
**Soil pH**	**0.296**	**0.122**	**2.418**	**0.020**
C/N ratio	0.234	0.130	1.794	0.080
Soil N	0.264	0.150	1.754	0.092
**Soil P**	**-0.367**	**0.147**	**-2.492**	**0.017**
Available P	0.016	0.144	0.115	0.909

Table S8. Results of linear mixed-effects models for soil fungal diversity (Shannon diversity) using plant community traits and soil properties as fixed factors separately. Plant community traits contained Masson pine dominance (PIV), ECM woody species dominance (EMIV), evergreen species dominance (EIV), all tree species dominance (TIV), plant PCoA1, plant PCoA2 and plant Shannon diversity. Soil properties included soil pH, maximum water holding capacity (MWHC), soil C/N ratio, soil N, soil P and available P. Impact factors in bold type were significant at $P < 0.05$.

**Impact factors**	**Estimate**	**SE**	**T**	***P***
Plant factors				
PIV	-0.039	0.147	-0.268	0.790
EMIV	0.242	0.154	1.574	0.123
EIV	0.062	0.147	0.423	0.674
TIV	-0.382	0.199	-1.916	0.062
**Plant PCoA1**	**-0.847**	**0.302**	**-2.798**	**0.008**
Plant PCoA2	0.114	0.158	0.726	0.471
Plant diversity	0.065	0.120	0.541	0.591
Soil factors				
MWNC	-0.092	0.195	-0.474	0.638
Soil pH	-0.041	0.126	-0.328	0.745
C/N ratio	0.065	0.154	0.421	0.677
Soil N	-0.049	0.194	-0.255	0.800
Soil P	-0.201	0.178	-1.129	0.266
Available P	0.069	0.142	0.490	0.627

Table S9. Results of linear mixed-effects models for soil pH using Masson pine dominance (PIV), ECM woody species dominance (EMIV), plant PCoA1, plant PCoA2 and plant Shannon diversity as fixed factors. Impact factor in bold type was significant at $P < 0.05$.

Impact factors	Estimate	SE	T	*P*
PIV	0.142	0.174	0.815	0.420
EMIV	0.173	0.133	1.301	0.202
Plant PCoA1	0.136	0.160	0.848	0.766
**Plant PCoA2**	**-0.518**	**0.133**	**-3.881**	**0.003**
Plant diversity	-0.056	0.148	-0.383	0.703

Table S10. Results of linear mixed-effects models for soil maximum water holding capacity (MWHC) using Masson pine dominance (PIV), ECM plant species dominance (EMIV), plant PCoA1, plant PCoA2 and plant Shannon diversity as fixed factors. Impact factor in bold type was significant at $P < 0.05$.

**Impact factors**	**Estimate**	**SE**	**T**	***P***
**PIV**	**-0.471**	**0.230**	**-2.050**	**0.047**
EMIV	0.034	0.208	0.164	0.871
Plant PCoA1	0.204	0.303	0.674	0.578
Plant PCoA2	0.101	0.200	0.508	0.618
Plant diversity	-0.161	0.253	-0.636	0.530

Table S11. Characteristics of the geographic location and woody plant community of the 44 Masson pine forest plots in southeastern China. PIV (importance values of Masson pine), Masson pine dominance; EIV (sum of importance values of all evergreen woody species), evergreen species dominance; TIV (sum of importance values of tree species without including shrubs), tree species dominance.

Plot ID	County location	Latitude (N)	Longitude (E)	PIV	EIV	TIV	Plant species richness	Plant Shannon diversity
C1	Chun'an	29°30′58″	118°49′42″	0.22	0.68	0.41	11.6	2.00
C2	Chun'an	29°30′29″	118°49′22″	0.23	0.87	0.45	13.2	2.10
C3	Chun'an	29°30′46″	118°49′35″	0.31	0.77	0.47	10.0	1.81
C4	Chun'an	29°30′37″	118°48′56″	0.27	0.82	0.46	13.0	2.04
C5	Chun'an	29°31′47″	118°55′21″	0.37	0.95	0.41	7.4	1.56
C6	Chun'an	29°30′24″	118°49′38″	0.20	0.72	0.47	11.6	2.01
C7	Chun'an	29°31′30″	118°55′48″	0.19	0.81	0.27	13.4	2.24
C8	Chun'an	29°32′11″	118°55′55″	0.25	0.79	0.37	12.4	2.07
C9	Chun'an	29°32′28″	118°53′00″	0.25	0.79	0.40	11.0	2.02
C10	Chun'an	29°29′26″	118°41′30″	0.19	0.72	0.34	12.2	1.98
C11	Chun'an	29°29′28″	118°41′33″	0.24	0.69	0.42	17.0	2.28
C12	Chun'an	29°29′33″	118°41′22″	0.13	0.64	0.34	12.0	2.17
C13	Chun'an	29°31′22″	118°58′09″	0.27	0.78	0.42	10.0	1.91
C14	Chun'an	29°31′19″	118°58′17″	0.23	0.79	0.42	10.4	2.03
C15	Chun'an	29°31′12″	118°57′37″	0.31	0.84	0.46	8.0	1.66
C16	Chun'an	29°31′07″	118°57′39″	0.26	0.76	0.44	9.0	1.91
C17	Chun'an	29°33′51″	118°59′09″	0.33	0.78	0.46	16.6	2.17
S1	Suichang	28°36′20″	118°56′08″	0.39	0.71	0.81	7.2	1.74
S2	Suichang	28°36′38″	118°57′21″	0.32	0.83	0.86	7.6	1.70
S3	Suichang	28°36′48″	118°57′29″	0.35	0.84	0.78	6.8	1.55
S4	Suichang	28°36′47″	118°57′29″	0.58	0.95	0.82	6.0	1.26
S5	Suichang	28°37′00″	119°27′22″	0.21	0.81	0.59	17.5	2.40
S6	Suichang	28°37′11″	119°27′37″	0.26	0.80	0.54	16.4	2.39
S7	Suichang	28°37′03″	119°27′27″	0.19	0.65	0.64	17.8	2.54
S8	Suichang	28°37′03″	119°26′58″	0.38	0.76	0.63	17.4	2.23
S9	Suichang	28°37′05″	119°26′48″	0.32	0.55	0.58	12.6	2.10
S10	Suichang	28°37′06″	119°26′48″	0.38	0.58	0.60	14.4	2.07
S11	Suichang	28°37′08″	119°26′48″	0.28	0.72	0.55	19.6	2.44
S12	Suichang	28°37′06″	119°26′50″	0.20	0.65	0.55	21.0	2.61
S13	Suichang	28°35′34″	119°27′39″	0.43	0.74	0.68	9.2	1.66
S14	Suichang	28°35′35″	119°27′39″	0.34	0.61	0.64	11.0	1.86
S15	Suichang	28°35′36″	119°27′40″	0.27	0.48	0.60	12.4	2.16
S16	Suichang	28°35′37″	119°27′40″	0.24	0.58	0.69	10.6	1.97
S17	Suichang	28°35′48″	119°27′56″	0.34	0.69	0.56	14.0	2.13
S18	Suichang	28°35′49″	119°28′02″	0.31	0.67	0.70	11.8	2.07
S19	Suichang	28°35′50″	119°28′00″	0.35	0.51	0.67	9.8	1.86
T1	Taishun	27°33′50″	119°41′56″	0.16	0.92	0.72	8.2	1.90
T2	Taishun	27°33′48″	119°41′57″	0.10	0.96	0.66	9.2	1.95
T3	Taishun	27°40′33″	119°46′55″	0.46	0.84	0.61	7.2	1.47
T4	Taishun	27°38′49″	119°46′50″	0.55	0.85	0.61	7.4	1.47
T5	Taishun	27°40′36″	119°46′48″	0.20	0.90	0.39	9.2	1.94
T6	Taishun	27°38′40″	119°46′41″	0.15	0.88	0.43	18.6	2.55

Plot ID	County location	Latitude (N)	Longitude (E)	PIV	EIV	TIV	Plant species richness	Plant Shannon diversity
T7	Taishun	27°38′45″	119°46′38″	0.34	0.91	0.44	12.0	1.94
T8	Taishun	27°40′17″	119°45′44″	0.16	0.87	0.63	14.4	2.32

Table S12. Mycorrhizal type, leaf habit and growth form of woody plants appeared in 44 plots. ECM, ectomycorrhizal type; Other, other mycorrhizal types, which mainly included arbuscular mycorrhizal type, ericoid mycorrhizal type and non-mycorrhizal type.

Plant species	Mycorrhizal type	Leaf habit	Growth form
Abelia chinensis	Other	Shrub	Deciduous
Acer cordatum	Other	Tree	Evergreen
Adina pilulifera	Other	Shrub	Evergreen
Adinandra millettii	Other	Shrub	Evergreen
Alangium kurzii	Other	Shrub	Deciduous
Albizia kalkora	Other	Tree	Deciduous
Alniphyllum fortunei	Other	Tree	Deciduous
Aralia elata	Other	Shrub	Deciduous
Broussonetia kazinoki	Other	Shrub	Deciduous
Callicarpa giraldii	Other	Shrub	Deciduous
Camellia fraterna	Other	Shrub	Evergreen
Camellia oleifera	Other	Shrub	Evergreen
Camellia sinensis	Other	Shrub	Evergreen
Camellia trichoclada	Other	Shrub	Evergreen
Carpinus polyneura	ECM	Tree	Deciduous
Castanea henryi	ECM	Tree	Deciduous
Castanea mollissima	ECM	Tree	Evergreen
Castanea seguinii	ECM	Shrub	Deciduous
Castanopsis carlesii	ECM	Tree	Evergreen
Castanopsis eyrei	ECM	Tree	Evergreen
Castanopsis jucunda	ECM	Tree	Evergreen
Castanopsis sclerophylla	ECM	Tree	Evergreen
Cerasus discoidea	Other	Shrub	Deciduous
Cerasus schneideriana	Other	Shrub	Deciduous
Chionanthus retusus	Other	Tree	Deciduous
Cinnamomum camphora	Other	Tree	Evergreen
Clerodendrum cyrtophyllum	Other	Shrub	Deciduous
Clerodendrum kaichianum	Other	Shrub	Deciduous
Corylopsis sinensis var. *calvescens*	Other	Shrub	Deciduous
Cryptomeria fortunei	Other	Tree	Evergreen
Cunninghamia lanceolata	Other	Tree	Evergreen
Cyclobalanopsis glauca	ECM	Tree	Evergreen
Dalbergia hupeana	Other	Tree	Deciduous
Dendropanax dentiger	Other	Shrub	Evergreen
Diospyros japonica	Other	Tree	Deciduous
Diospyros kaki var. *silvestris*	Other	Tree	Deciduous

Plant species	Mycorrhizal type	Leaf habit	Growth form
Diospyros morrisiana	Other	Tree	Evergreen
Diospyros tsangii	Other	Shrub	Deciduous
Diplospora dubia	Other	Shrub	Evergreen
Elaeocarpus decipiens	Other	Tree	Evergreen
Eurya hebeclados	Other	Shrub	Evergreen
Eurya muricata	Other	Shrub	Evergreen
Eurya rubiginosa var. attenuata	Other	Shrub	Evergreen
Euscaphis japonica	Other	Shrub	Deciduous
Ficus erecta	Other	Shrub	Deciduous
Ficus variolosa	Other	Shrub	Evergreen
Fraxinus insularis	Other	Tree	Deciduous
Gardenia jasminoides	Other	Shrub	Evergreen
Glochidion puberum	Other	Shrub	Deciduous
Ilex chinensis	Other	Tree	Evergreen
Ilex cornuta	Other	Shrub	Evergreen
Ilex crenata	Other	Shrub	Evergreen
Ilex elmerrilliana	Other	Shrub	Evergreen
Ilex ficoidea	Other	Tree	Evergreen
Ilex litseifolia	Other	Shrub	Evergreen
Ilex lohfauensis	Other	Shrub	Evergreen
Ilex pubescens	Other	Shrub	Evergreen
Ilex rotunda	Other	Tree	Evergreen
Ilex suaveolens	Other	Tree	Evergreen
Ilex triflora	Other	Shrub	Evergreen
Ilex wilsonii	Other	Tree	Evergreen
Itea chinensis	Other	Shrub	Evergreen
Itea omeiensis	Other	Shrub	Evergreen
Juniperus formosana	Other	Tree	Evergreen
Lindera aggregata	Other	Shrub	Evergreen
Lindera erythrocarpa	Other	Shrub	Deciduous
Lindera glauca	Other	Shrub	Deciduous
Lindera reflexa	Other	Shrub	Deciduous
Liquidambar formosana	ECM	大Tree	Deciduous
Lithocarpus glaber	ECM	Tree	Evergreen
Lithocarpus hancei	ECM	Tree	Evergreen
Litsea coreana var. *sinensis*	Other	Tree	Evergreen
Litsea cubeba	Other	Shrub	Deciduous
Loropetalum chinense	ECM	Shrub	Evergreen
Lyonia ovalifolia var. *hebecarpa*	Other	Shrub	Deciduous
Machilus grijsii	Other	Tree	Evergreen
Machilus thunbergii	Other	Tree	Evergreen
Machilus velutina	Other	Tree	Evergreen
Mallotus apelta	Other	Shrub	Deciduous
Malus leiocalyca	Other	Tree	Deciduous
Myrica rubra	Other	Tree	Evergreen
Ormosia henryi	Other	Tree	Evergreen

Plant species	Mycorrhizal type	Leaf habit	Growth form
Osmanthus cooperi	Other	Shrub	Evergreen
Osmanthus fragrans	Other	Shrub	Evergreen
Pertusadina hainanensis	Other	Shrub	Evergreen
Phoebe bournei	Other	Tree	Evergreen
Phoebe chekiangensis	Other	Tree	Evergreen
Photinia beauverdiana	Other	Shrub	Deciduous
Photinia glabra	Other	Shrub	Evergreen
Photinia hirsuta	Other	Shrub	Deciduous
Photinia parvifolia	Other	Shrub	Deciduous
Photinia schneideriana	Other	Shrub	Deciduous
Pinus massoniana	ECM	Tree	Evergreen
Pinus taiwanensis	ECM	Tree	Evergreen
Platycarya strobilacea	ECM	Shrub	Deciduous
Premna microphylla	Other	Shrub	Deciduous
Pyrenaria microcarpa	Other	Shrub	Evergreen
Quercus acutissima	ECM	Tree	Deciduous
Quercus fabri	ECM	Tree	Deciduous
Quercus phillyreoides	ECM	Shrub	Evergreen
Quercus serrata var. *brevipetiolata*	ECM	Tree	Deciduous
Rhamnus crenata	Other	Shrub	Deciduous
Rhamnus wilsonii	Other	Shrub	Deciduous
Rhaphiolepis indica	Other	Shrub	Evergreen
Rhododendron championiae	Other	Shrub	Evergreen
Rhododendron mariesii	Other	Shrub	Deciduous
Rhododendron ovatum	Other	Shrub	Evergreen
Rhododendron simsii	Other	Shrub	Deciduous
Rhus chinensis	Other	Shrub	Deciduous
Rubus chingii	Other	Shrub	Deciduous
Schima superba	Other	Tree	Evergreen
Schoepfia jasminodora	Other	Shrub	Deciduous
Styrax confusus	Other	Shrub	Deciduous
Styrax dasyanthus	Other	Shrub	Deciduous
Styrax faberi	Other	Shrub	Deciduous
Styrax odoratissimus	Other	Shrub	Deciduous
Symplocos anomala	Other	Shrub	Evergreen
Symplocos laurina	Other	Shrub	Evergreen
Symplocos paniculata	Other	Shrub	Deciduous
Symplocos setchuensis	Other	Shrub	Evergreen
Symplocos stellaris	Other	Shrub	Evergreen
Symplocos sumuntia	Other	Shrub	Evergreen
Syzygium buxifolium	Other	Shrub	Evergreen
Tarenna mollissima	Other	Shrub	Deciduous

Plant species	Mycorrhizal type	Leaf habit	Growth form
Ternstroemia gymnanthera	Other	Tree	Evergreen
Tetradium glabrifolium	Other	Tree	Deciduous
Toxicodendron succedaneum	Other	Tree	Deciduous
Toxicodendron sylvestre	Other	Tree	Deciduous
Toxicodendron trichocarpum	Other	Shrub	Deciduous
Triadica cochinchinensis	Other	Shrub	Deciduous
Triadica sebifera	Other	Tree	Deciduous
Vaccinium bracteatum	Other	Shrub	Evergreen
Vaccinium carlesii	Other	Shrub	Evergreen
Vaccinium mandarinorum	Other	Shrub	Evergreen
Vcmicia Montana	Other	Tree	Deciduous
Vernicia fordii	Other	Tree	Deciduous
Viburnum dilatatum	Other	Shrub	Deciduous
Viburnum erosum	Other	Shrub	Deciduous
Viburnum sempervirens	Other	Shrub	Evergreen
Viburnum setigerum	Other	Shrub	Deciduous
Vitex negundo var. *cannabifolia*	Other	Shrub	Deciduous
Wikstroemia monnula	Other	Shrub	Deciduous
Xylosma congesta	Other	Shrub	Evergreen
Yulania cylindrica	Other	Tree	Deciduous

Table S13. The first two axes values of Principal component analysis (PC1 and PC2) for 14 environmental factors. Environmental factors contained PIV, EMIV, TIV, EIV, plant basal area, plant density, soil pH, soil bulk density, MWHC, C/N ratio, soil P, NH_4^+-N, NO_3^--N and available P. PIV (importance values of Masson pine), Masson pine dominance; EIV (sum of importance values of all evergreen woody species), evergreen species dominance; TIV (sum of importance values of all tree species), tree species dominance; MWHC, soil maximum water holding capacity.

Plot ID	PC1	PC2
C1	-0.46442	0.15495
C2	0.080347	0.64516
C3	0.609012	-0.07315
C4	0.426301	0.98412
C5	1.456261	0.07008
C6	-0.21114	0.06996
C7	-1.09328	1.02902
C8	-0.67641	0.64911
C9	-0.44333	0.31395
C10	-0.69599	-2.23728
C11	-0.6981	-0.68966
C12	-1.51169	-0.67098
C13	0.577589	0.02322
C14	0.484435	0.20818
C15	1.463294	-0.3729
C16	0.666605	0.02685
C17	0.030514	-0.13064
S1	1.48974	-0.61393
S2	1.362375	0.75859
S3	0.990328	0.64704
S4	1.533768	-0.60938
S5	-0.04279	0.51645
S6	-0.13288	0.75542
S7	0.020557	0.34978
S8	-0.09509	0.93178
S9	-0.88998	0.58912
S10	-0.68557	0.29038
S11	-0.64769	0.86799
S12	-0.92221	0.94208
S13	0.045431	-0.46767
S14	-0.35749	-0.37777
S15	-0.60272	-0.11347
S16	-0.2995	0.02697
S17	-0.18414	-1.56205
S18	-0.27018	-0.77647
S19	-0.31196	-2.15485
T1	0.757602	0.14169
T2	0.736867	0.53201
T3	-0.02302	-0.80856

Plot ID	PC1	PC2
T4	0.37058	-0.24499
T5	-0.38032	0.28443
T6	-0.95911	0.32757
T7	-0.50847	0.36612
T8	0.005872	-0.59827
